# Supplementary material for: Accurate Mediterranean Sea forecasting via graph-based deep learning
Source: Sci Rep. 2025 Dec 6;15:45051. doi: 10.1038/s41598-025-31177-w (PMC12748638; doi:10.1038/s41598-025-31177-w)
Supplement: Supplementary file 1 — Supplementary Information. [file 41598_2025_31177_MOESM1_ESM.pdf]

# Supplementary Materials

## Accurate Mediterranean Sea forecasting via graph-based deep learning

Daniel Holmberg, Emanuela Clementi, Italo Epicoco, Teemu Roos

## Contents

**A Data details** **2**
- A.1 Region considered . . . 2
- A.2 Variables considered . . . 2
- A.3 Satellite data . . . 2

**B Model Details** **4**

**C Evaluation metrics** **5**

**D Additional results** **6**
- D.1 In-situ evaluation . . . 6
- D.2 Analysis evaluation . . . 7
- D.3 Vertical error profiles . . . 11
- D.4 Effect of atmospheric forcing . . . 13
- D.5 Effect of training period . . . 16

**E Example forecasts** **19**

# A Data details

## A.1 Region considered

The domain used in this study covers the Mediterranean Sea, with horizontal resolution of 1/24°, from the surface down to 200 m depth, and includes two lateral open boundary regions where external forcing is applied. These boundaries allow the model to account for the inflow and outflow of water with neighboring seas. The first open boundary is located at the western edge of the domain, near the Strait of Gibraltar connecting to the Atlantic Ocean, and includes all grid cells west of longitude 5.2°W. The second open boundary is situated in the northeastern corner of the domain, corresponding to the Dardanelles Strait connecting to the Black Sea, and spans the region bounded by latitudes 39.9°N to 40.4°N and longitudes 25.9°E to 26.4°E. These regions are highlighted in red in Figure S1, overlaid on the bathymetry map of the Mediterranean Sea.

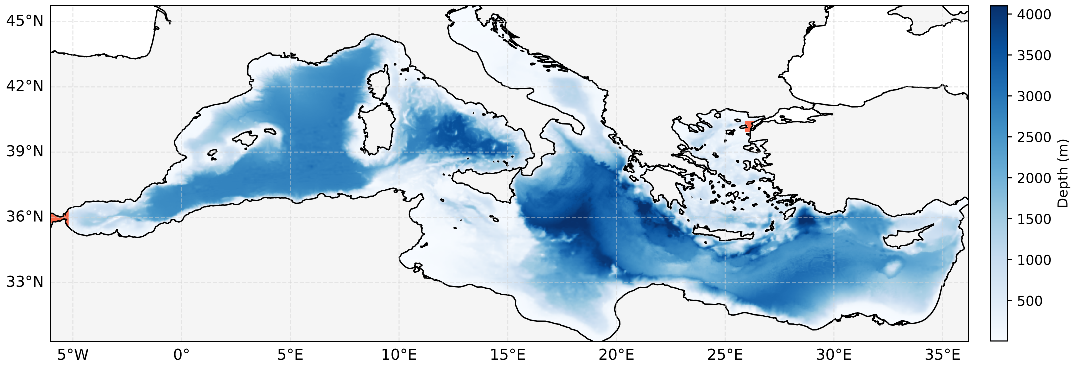


**Fig. S1** Bathymetry of the Mediterranean Sea domain used in SeaCast, with forcing regions highlighted in red. The western boundary at the Strait of Gibraltar and the eastern boundary at the Dardanelles Strait are designated as open boundaries, where boundary conditions are applied to account for water exchange with adjacent seas.

The minimum rectangle in which the Mediterranean Sea fits at the current resolution is 371 by 1013 grid cells. This is a total of 375 823 cells, whereas the actual sea surface of the Mediterranean only has $N = 144\ 990$ cells, or 45% of the total number. Only this subset of grid cells has to be processed by SeaCast. The complete data grid is shown in Figure S1. Single-level features, forcing features, and static fields all cover the surface area, whereas the simulated currents, temperature and salinity have values on several vertical levels.

## A.2 Variables considered

The sea physics dataset used to train and evaluate SeaCast consists of dynamic variables, static fields, and atmospheric forcing inputs. Table S1 provides a detailed overview of all included features. The core variables represent the three-dimensional state of the ocean, including currents, temperature, salinity provided at 18 vertical levels, as well as SSH (73 predicted fields in total). The bathymetry and mean dynamic topography static fields are used to define the spatial structure of the ocean domain. Finally, surface atmospheric forcing variables including wind stress, 2-meter temperature, and MSLP are incorporated to drive the ocean dynamics, along with cyclical encodings of time of year to account for seasonal variability.

## A.3 Satellite data

Predicted SST and SLA are compared also to satellite observations. SST is fairly straightforward to compare on the satellite L3S SST grid, but for SLA there is a bit of a procedure

**Table S1** Summary of all variables, static fields, and forcing features in the Mediterranean Sea physics dataset.

| | Unit | Vertical Level |
|---|---|---|
| Variables | | |
| Zonal sea water velocity | m/s | 18 depths |
| Meridional sea water velocity | m/s | 18 depths |
| Sea water salinity | psu | 18 depths |
| Sea water potential temperature | °C | 18 depths |
| Sea surface height above geoid | m | Sea surface |
| Static fields | | |
| Sea floor depth below geoid | m | Sea floor |
| Mean dynamic topography | m | Sea surface |
| Latitude | ° | - |
| Longitude | ° | - |
| Forcing | | |
| Zonal wind stress | Pa | Sea surface |
| Meridional wind stress | Pa | Sea surface |
| 2-meter temperature | °C | Sea surface |
| Mean sea level pressure | Pa | Sea surface |
| Sine of time of year | - | - |
| Cosine of time of year | - | - |

to align it with L3 measurements. To compute the observed SLA used in evaluation, we take the filtered SLA satellite data and apply dynamic atmospheric correction, ocean tide correction, and internal tide correction. These additional terms account for physical processes that influence sea level but are not captured directly in the raw altimeter measurements.

In contrast, the sea level forecasts from MedFS and SeaCast are provided as absolute SSH relative to the geoid. To make this comparable to observed SLA, we subtract the model's mean dynamic topography, effectively aligning the model output to a time-mean reference sea level similar to the satellite product, which uses the 1993–2012 period as its climatological baseline.

Each satellite track is split into ocean-only segments by applying a sea mask. Tracks are divided wherever land is encountered, and only segments containing a minimum number of four valid ocean points are retained for evaluation. Finally, to minimize any residual bias in comparisons along individual satellite tracks, we remove the average SLA value from each track in both the model forecasts and the observations.

# B Model Details

SeaCast operates on a multi-resolution graph representation of the Mediterranean Sea, coarser than the original grid, enabling the model to capture ocean dynamics across multiple spatial scales, while being computationally feasible to process on. Input variables are mapped onto a three-layer hierarchical graph, illustrated in Figure S2. Each graph layer captures ocean variability at a distinct resolution, allowing the model to learn both short and long range interactions that are useful for predicting future sea states.

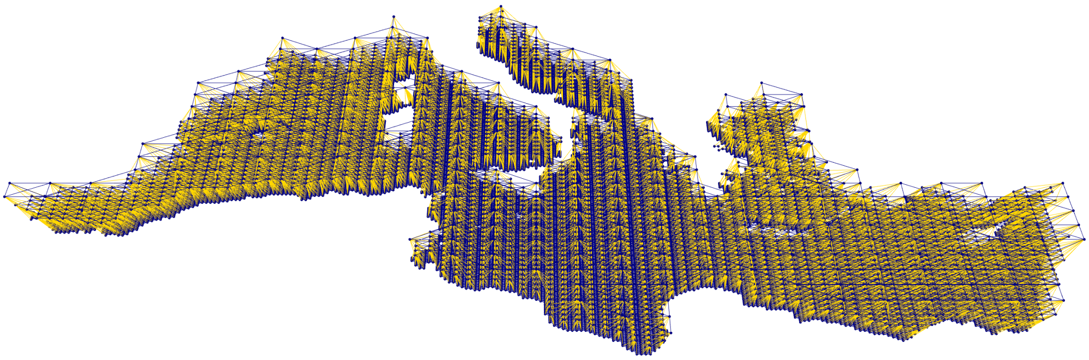

**Fig. S2** Ocean variables are encoded onto a hierarchical mesh of the Mediterranean Sea shown here. Each layer has a different resolution allowing for interactions at different scales between observables.

Each node is connected via bidirectional edges to its immediate neighbors—horizontally, vertically, and diagonally. This connectivity pattern is repeated across three resolutions, with the spacing between nodes tripling at each successive level. Specifically, a node at resolution level $h$ is placed at the center of a $3 \times 3$ patch of nodes at resolution $h - 1$.

Table S2 summarizes the structure of the full graph used in SeaCast, including the number of nodes and edges at each layer, as well as the mapping components that connect the physical grid, mesh, and graph representations. The base layer, $\mathcal{G}_0$, corresponds to the finest resolution in the mesh. Two additional layers, $\mathcal{G}_1$ and $\mathcal{G}_2$, represent increasingly coarser resolutions, allowing for efficient message passing over larger spatial extents.

**Table S2** Number of nodes and edges in the sea graph.

| Graph | Nodes | Edges |
|---|---|---|
| $\mathcal{G}_0$ | 22677 | 174007 |
| $\mathcal{G}_{0,1}/\mathcal{G}_{1,0}$ | - | 22677 |
| $\mathcal{G}_1$ | 2515 | 18206 |
| $\mathcal{G}_{1,2}/\mathcal{G}_{2,1}$ | - | 2515 |
| $\mathcal{G}_2$ | 272 | 1610 |
| Mesh | 25464 | 219015 |
| $\mathcal{G}_{G2M}$ | - | 542271 |
| $\mathcal{G}_{M2G}$ | - | 579960 |
| Grid | 144990 | - |

Inter-layer connections, such as $\mathcal{G}_{0,1}$ and $\mathcal{G}_{1,2}$, define bidirectional edges that facilitate hierarchical information flow between levels. Additionally, SeaCast includes mappings between the physical simulation grid and the mesh graph, denoted $\mathcal{G}_{G2M}$ (grid-to-mesh) and $\mathcal{G}_{M2G}$ (mesh-to-grid), which enable the model to interface with the ocean data grid.

## C Evaluation metrics

***Root mean squared error (RMSE).*** For each lead time $t$, variable $i$, and depth level $l$ the RMSE is defined as:

$$\mathrm{RMSE}_{t,i,l} = \sqrt{\frac{1}{T_{\text{eval}}} \sum_{t_0=1}^{T_{\text{eval}}} \frac{1}{N_l} \sum_{v=1}^{N_l} a_v \left( \hat{X}_{v,i}^{t_0+t} - X_{v,i}^{t_0+t} \right)^2}. \tag{1}$$

Here, $t_0$ indexes forecast initialization times up to $T_{\text{eval}}$; $v$ indexes valid grid nodes at depth level $l$ up to $N_l$; $a_v$ is the cell-area weight (normalized to unit mean); and $\hat{X}$ and $X$ denote the predicted and observed values, respectively.

***Root mean squared error (RMSE), depth-averaged.*** For each lead time $t$ and variable $i$, the RMSE is defined as:

$$\mathrm{RMSE}_{t,i} = \sqrt{\frac{1}{T_{\text{eval}}} \sum_{t_0=1}^{T_{\text{eval}}} \frac{1}{L_i} \sum_{l=1}^{L_i} \frac{1}{N_l} \sum_{v=1}^{N_l} a_v \left( \hat{X}_{v,i}^{t_0+t} - X_{v,i}^{t_0+t} \right)^2}. \tag{2}$$

Here, $l$ indexes the vertical levels associated with variable $i$ up to $L_i$, and the other variables are as defined above.

***Root mean squared error (RMSE), per location.*** We evaluate model outputs on observed SST also by breaking down RMSE per location. The spatial RMSE is calculated for lead time $t$, variable $i$, and grid point $v$ as:

$$\mathrm{RMSE}_{t,i,v} = \sqrt{\frac{1}{T_{\text{eval}}} \sum_{t_0=1}^{T_{\text{eval}}} \left( \hat{X}_{v,i}^{t_0+t} - X_{v,i}^{t_0+t} \right)^2}. \tag{3}$$

***Heidke skill score (HSS).*** We evaluate the models' ability to detect temperature extremes for each lead time using The Heidke Skill Score (HSS) derived from the contingency table consisting of true positives (TP), false positives (FP), false negatives (FN), and true negatives (TN). HSS compares forecast accuracy to that expected by chance. It is defined by:

$$\mathrm{HSS} = \frac{2\,(TP \cdot TN - FP \cdot FN)}{(TP + FN)(FN + TN) + (TP + FP)(FP + TN)}. \tag{4}$$

An HSS of 1 indicates a perfect forecast, 0 indicates no better than chance, and negative values indicate performance worse than a random forecast.

***Anomaly Correlation Coefficient (ACC), depth-averaged.*** For each lead time $t$ and multi-level variable $i$, we define the forecast and analysis anomalies as:

$$A_{v,i}^{t_0+t} = \hat{X}_{v,i}^{t_0+t} - C_{v,i}^{t_0+t}, \quad B_{v,i}^{t_0+t} = X_{v,i}^{t_0+t} - C_{v,i}^{t_0+t}. \tag{5}$$

The anomaly correlation coefficient is then computed as:

$$\mathrm{ACC}_{t,i} = \frac{1}{T_{\text{eval}}} \sum_{t_0=1}^{T_{\text{eval}}} \frac{\frac{1}{L_i} \sum_{l=1}^{L_i} \sum_{v=1}^{N_l} a_v A_{v,i}^{t_0+t} B_{v,i}^{t_0+t}}{\sqrt{\left[ \frac{1}{L_i} \sum_{l=1}^{L_i} \sum_{v=1}^{N_l} a_v \left( A_{v,i}^{t_0+t} \right)^2 \right] \left[ \frac{1}{L_i} \sum_{l=1}^{L_i} \sum_{v=1}^{N_l} a_v \left( B_{v,i}^{t_0+t} \right)^2 \right]}}. \tag{6}$$

Here, $C_{v,i}^{t_0+t}$ is the daily climatology at grid point $v$ and time $t_0 + t$, and the other variables are as defined above. ACC hence measures the correlation between the deviation of the prediction and ground truth from daily climatology.

# D Additional results

## D.1 In-situ evaluation

To assess forecasted depth-wise variables on ground-truth beyond gridded analysis data, we compare them against quality controlled in-situ measurements from the Mediterranean Sea. The in-situ data is sourced from the CMEMS In Situ Thematic Assembly Centre, which compiles observations from a broad network of national and international monitoring systems. To ensure consistency with the model output, the observations are spatially and temporally bin-averaged, following the SeaCast simulation grid, to produce daily mean values. Forecast fields are interpolated bilinearly to the latitude–longitude coordinates of the observations and linearly in the vertical to match the measured depths.

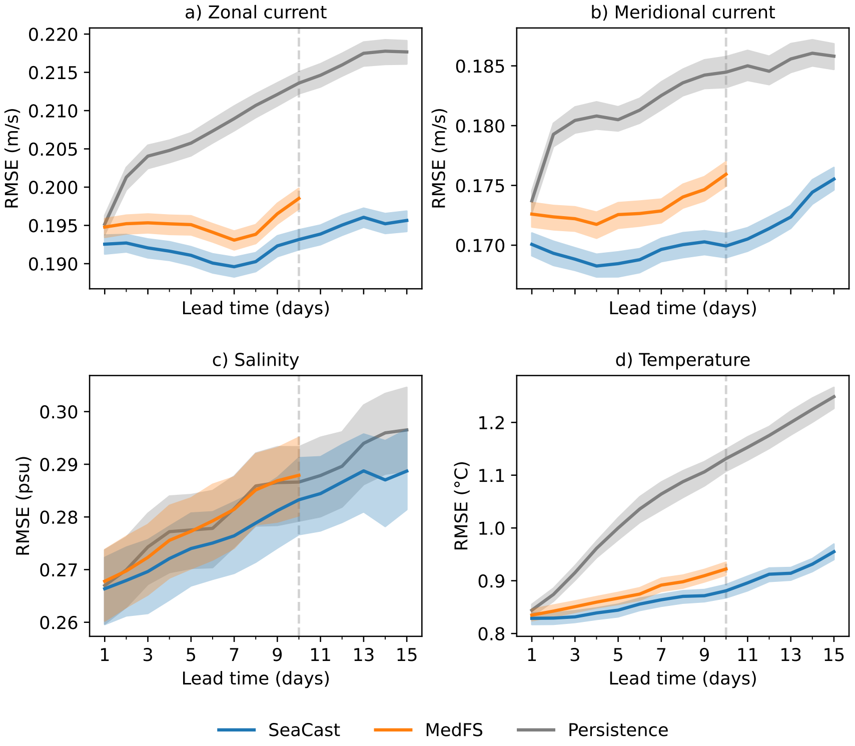


**Fig. S3** RMSE as a function of lead time for SeaCast forecasts compared to in-situ measurements. Results are shown for zonal and meridional currents, salinity, and temperature. The shading corresponds to the 50% confidence intervals estimated via bootstrapping.

## D.2 Analysis evaluation

Here, model forecasts are evaluated against analysis fields, using both depth-averaged ACC and RMSE at every other forecasted depth level. To account for the impact of the weekly data assimilation cycle, we also compute errors separately for forecasts initialized on the best performing day of the week, which is on Tuesday. Given the sizeable improvements we see below, these results make the case for performing assimilation more often, or at least aim to do so in the future. Zonal and meridional currents behave so similarly that only the former is included for the RMSE evaluation.

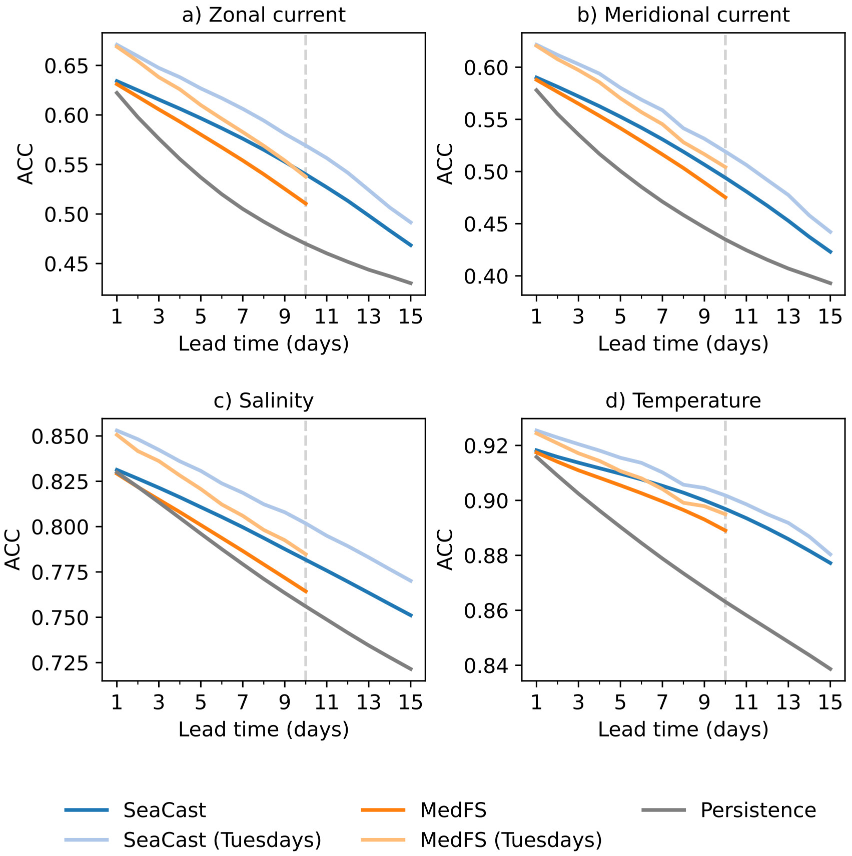


**Fig. S4** ACC versus lead time for SeaCast, MedFS, and a persistence baseline. Additional SeaCast and MedFS results from Tuesday initializations are included to account for peak performance due to weekly data assimilation.

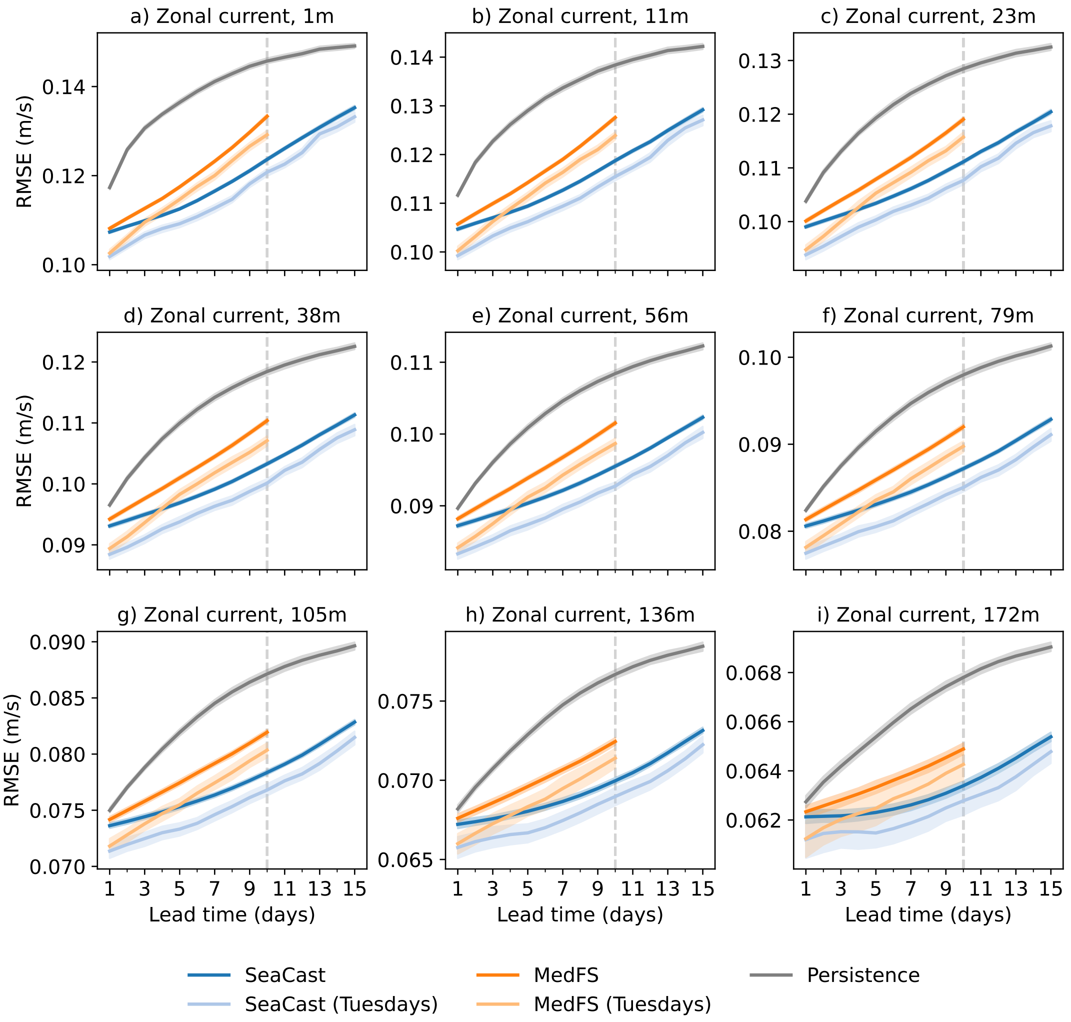


**Fig. S5** RMSE of zonal currents versus lead time across depth levels. Each subplot corresponds to a different depth. Comparisons include SeaCast, MedFS, and persistence. Additional SeaCast and MedFS results from Tuesday initializations are included to account for peak performance due to weekly data assimilation. The shading corresponds to the 50% confidence intervals estimated via bootstrapping.

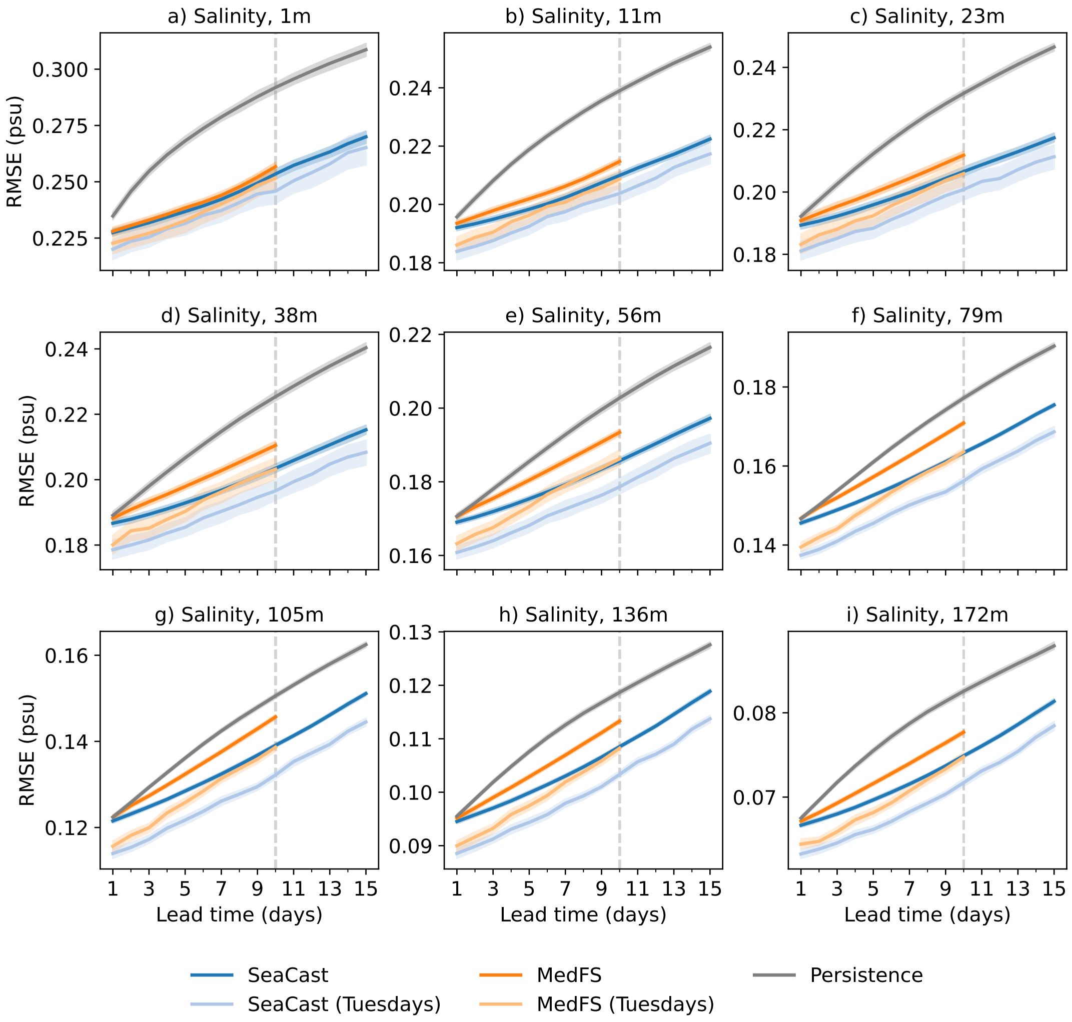


**Fig. S6** RMSE of salinity versus lead time across depth levels. Each subplot represents a different depth, comparing SeaCast, MedFS, and persistence. Additional SeaCast and MedFS results from Tuesday initializations are included to account for peak performance due to weekly data assimilation. The shading corresponds to the 50% confidence intervals estimated via bootstrapping.

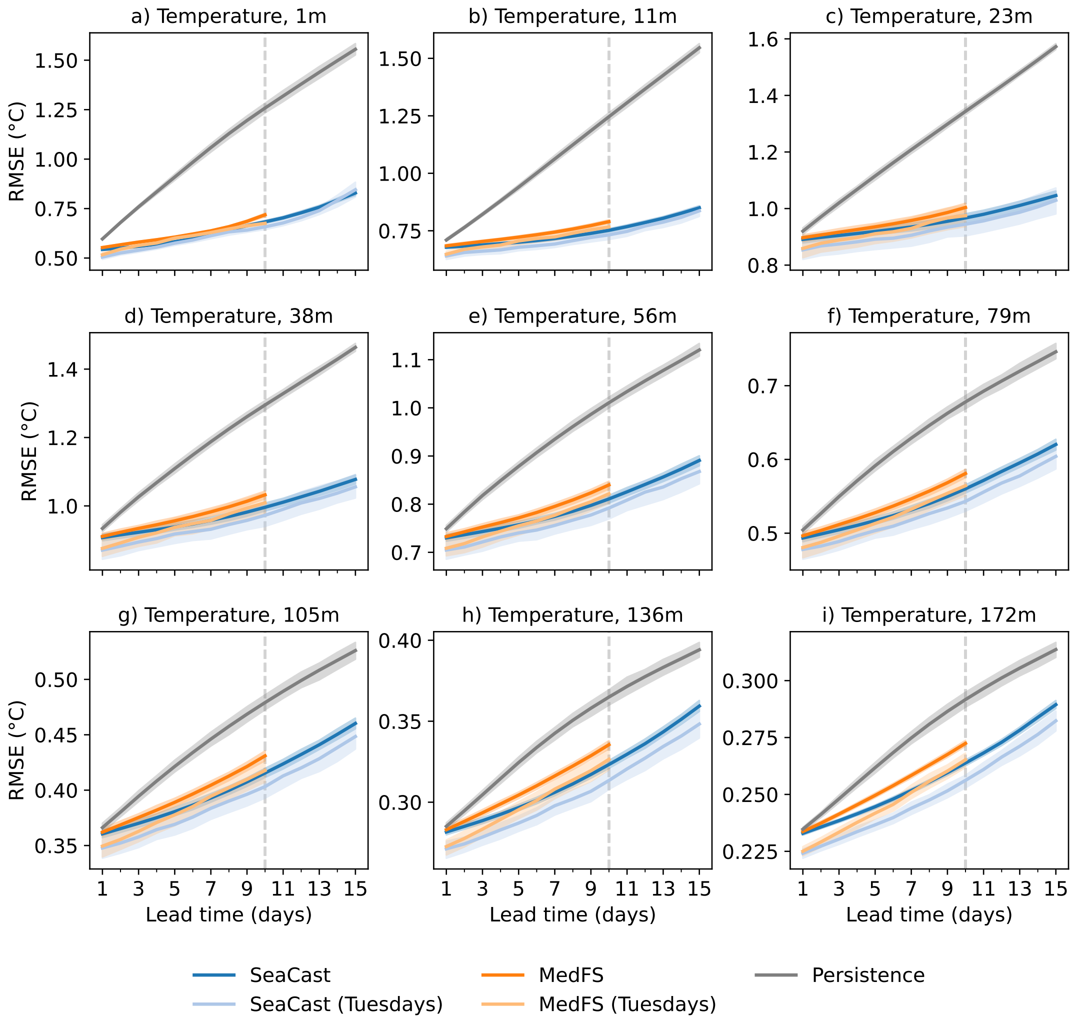


**Fig. S7** RMSE of temperature versus lead time across depth levels. Each subplot corresponds to a distinct depth, showing performance of SeaCast, MedFS, and persistence. Additional SeaCast and MedFS results from Tuesday initializations are included to account for peak performance due to weekly data assimilation. The shading corresponds to the 50% confidence intervals estimated via bootstrapping.

## D.3 Vertical error profiles

To better understand how forecast skill varies with depth, we analyze RMSE profiles across the vertical column for key ocean variables. These profiles are averaged over lead times only to reveal depth-dependent behavior. SeaCast initialized from the best available analysis fields serves as a lower bound on forecast error, while a persistence baseline provides an upper bound. Zonal and meridional currents behave so similarly that only the former is included here.

As seen in Figures S9 and S10, temperature, and salinity to a small extent, exhibit a bump in RMSE between approximately 10 and 60 meters depth. This depth range often corresponds to the base of the mixed layer during late summer-autumn seasons (which comprise a majority of the evaluation set), a zone prone to rapid transitions in stratification driven by atmospheric forcing or mesoscale variability, dynamics that remain challenging to capture accurately in simulations. Figure S8 shows that RMSE for zonal currents does not exhibit a similar bump, indicating that errors in current forecasts are more vertically uniform and less sensitive to stratification-related biases compared to scalar fields like temperature and salinity.

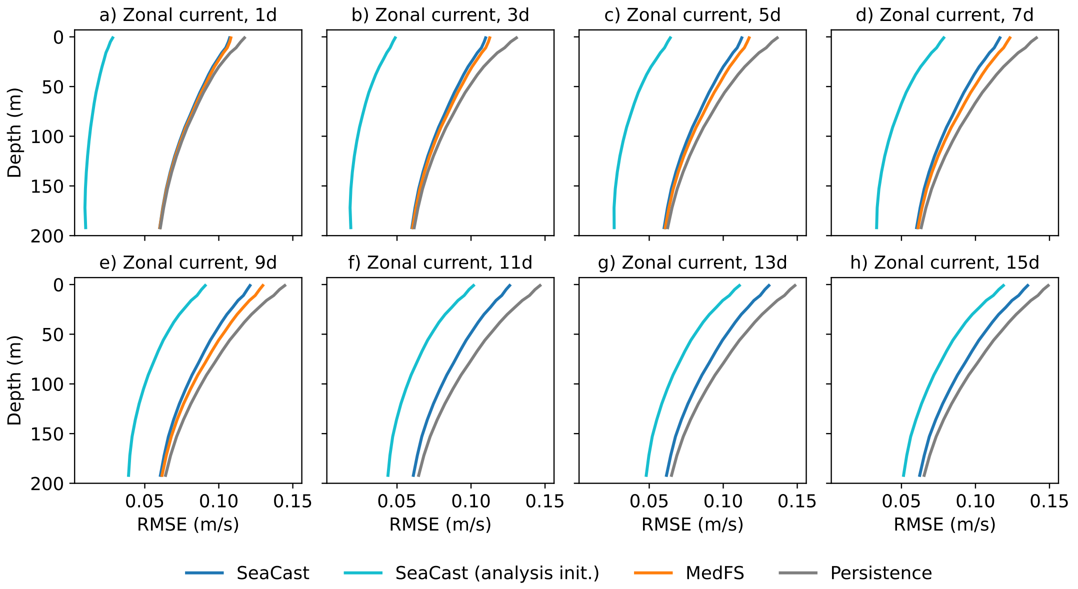


**Fig. S8** Vertical profile of RMSE for zonal currents averaged across lead times. SeaCast initialized from analysis fields and a persistence model are included to show upper and lower performance bounds across depths.

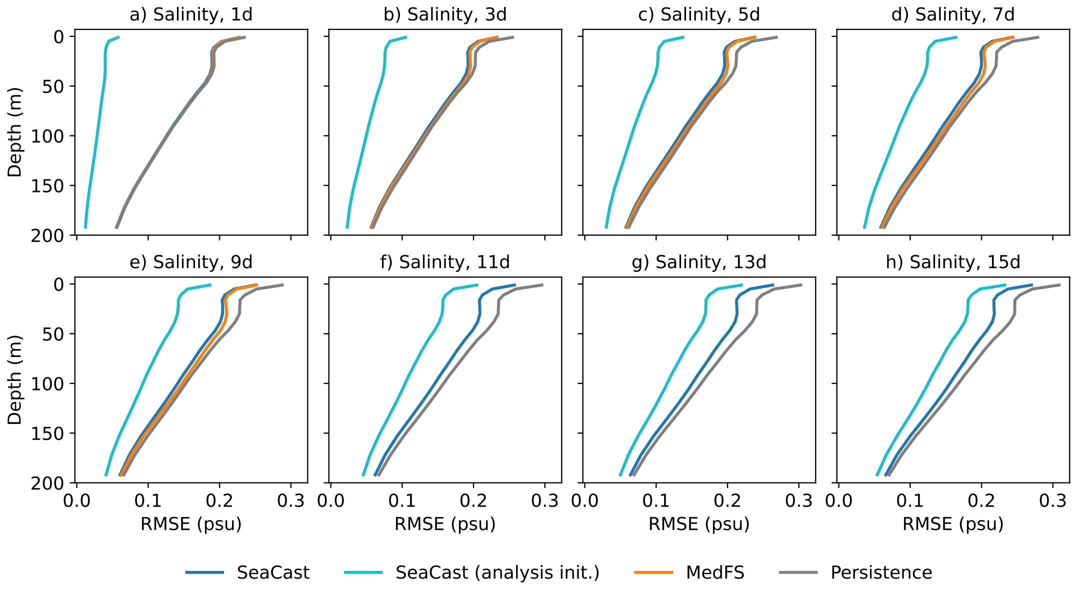


**Fig. S9** Vertical profile of RMSE for salinity, averaged over all lead times. SeaCast initialized from analysis fields and a persistence model are included to show upper and lower performance bounds across depths.

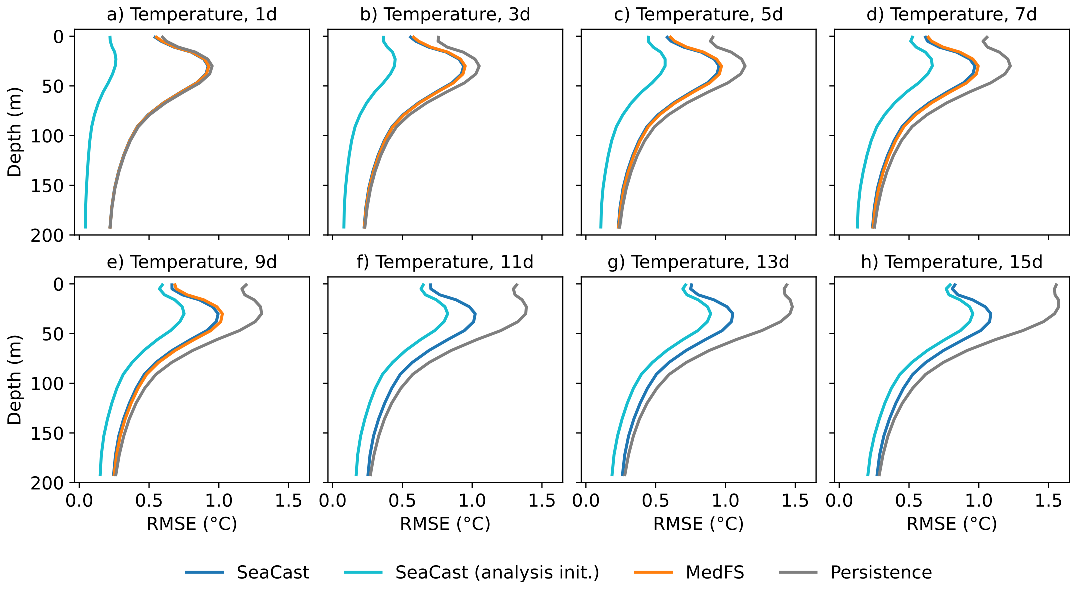


**Fig. S10** Vertical profile of RMSE for temperature, averaged across lead times. SeaCast initialized from analysis fields and a persistence model are included to show upper and lower performance bounds across depths.

## D.4 Effect of atmospheric forcing

To examine the sensitivity of SeaCast to surface atmospheric inputs, we perform a series of ablation studies in which individual atmospheric fields are permuted during inference. The normalized RMSE differences reveal the relative importance of each forcing variable and their influence on forecast accuracy at different depths and lead times. Zonal and meridional currents behave so similarly that only the former is included here. We can see a clear trend of the atmospheric forcing being more impactful closer to the sea surface in the plots below.

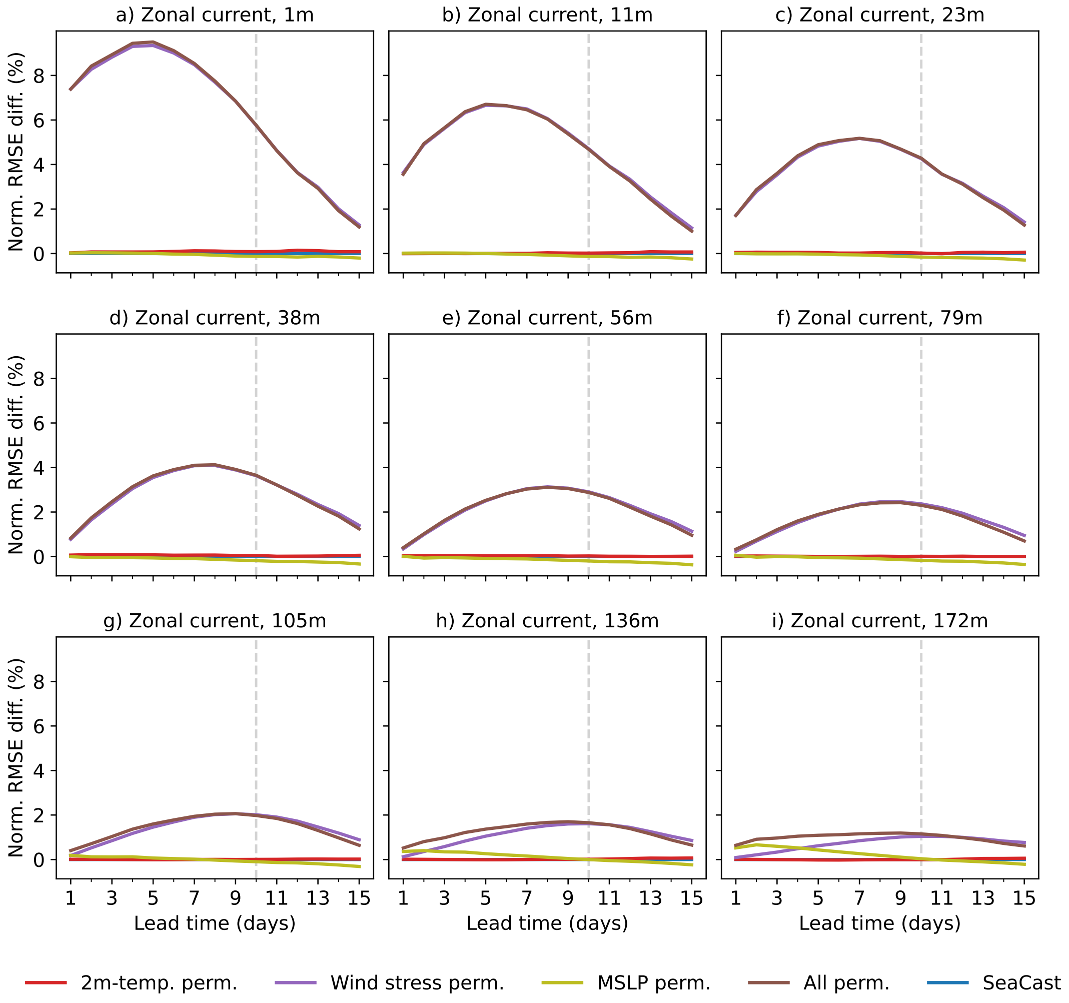


**Fig. S11** Normalized RMSE difference for zonal currents per lead time across depths. Results show the impact of permuting individual atmospheric forcing fields relative to the original SeaCast configuration.

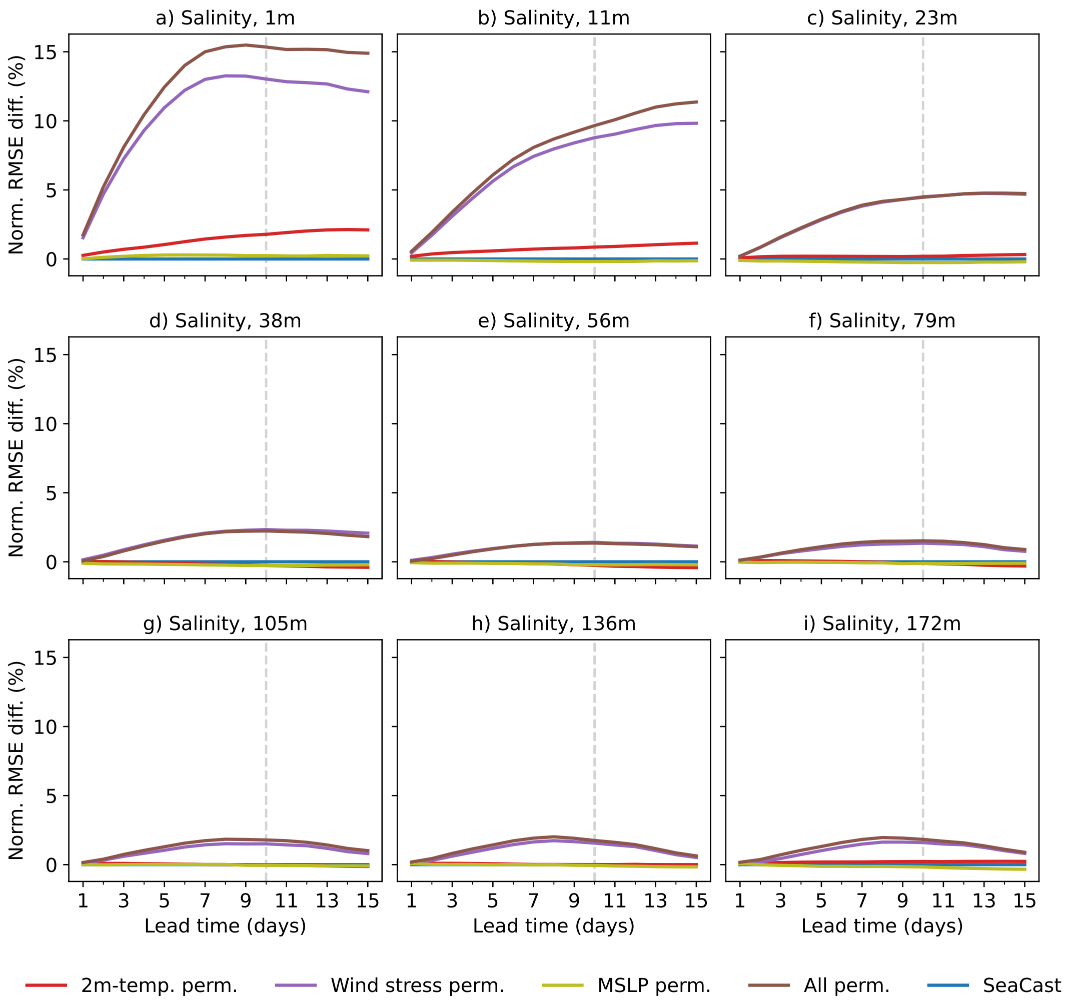


**Fig. S12** Normalized RMSE difference for salinity per lead time across depths. Comparisons show the impact of permuted atmospheric forcings versus the unperturbed SeaCast baseline.

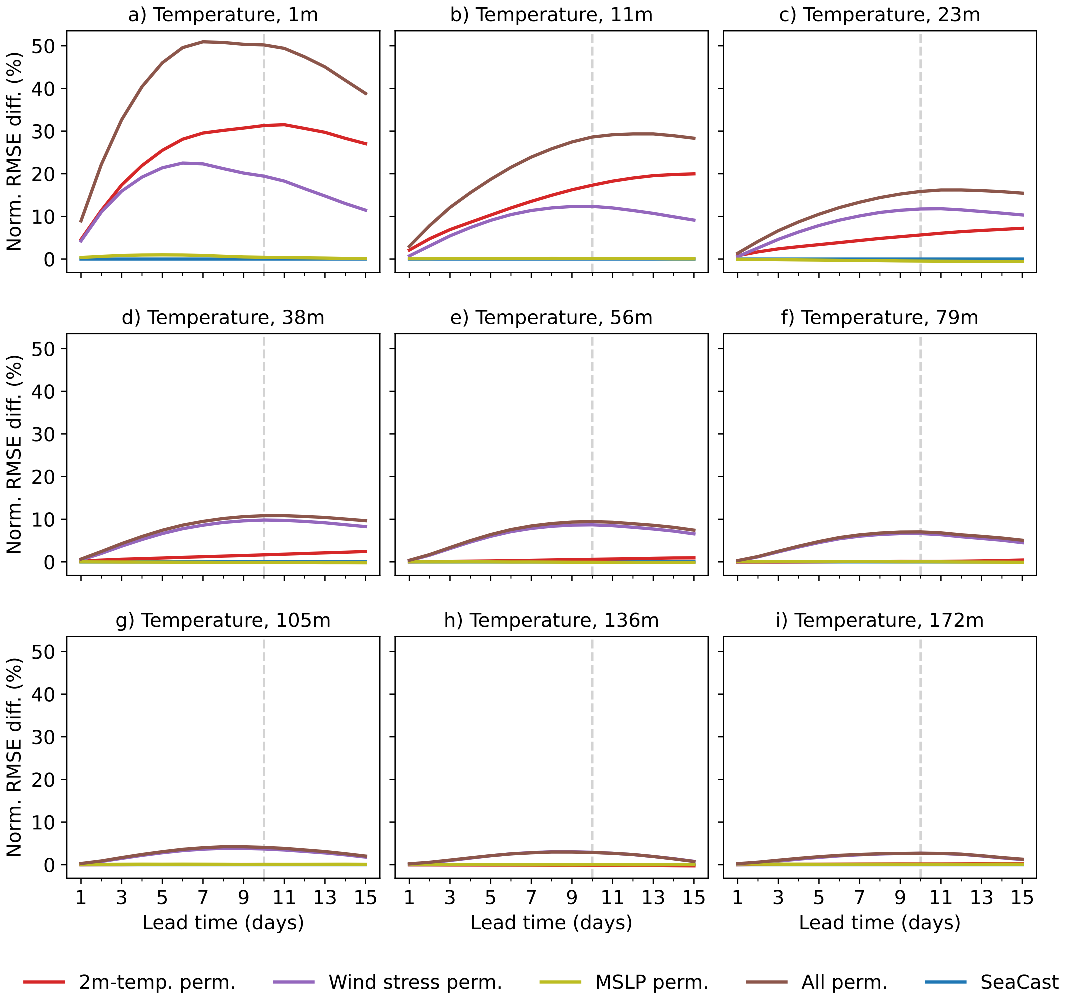


**Fig. S13** Normalized RMSE difference for temperature per lead time across depths. Each line shows the effect of permuting one atmospheric variable compared to the original SeaCast model.

## D.5 Effect of training period

Here, we explore how extending the training period affects forecast performance by comparing SeaCast models trained on 35 years and 8 years of reanalysis data (w/o finetuning) to models that are further finetuned on 2 years of analysis data. The forecasts are benchmarked against the persistence model using the normalized RMSE difference as skill metric for each lead time. Zonal and meridional currents behave so similarly that only the former is included here.

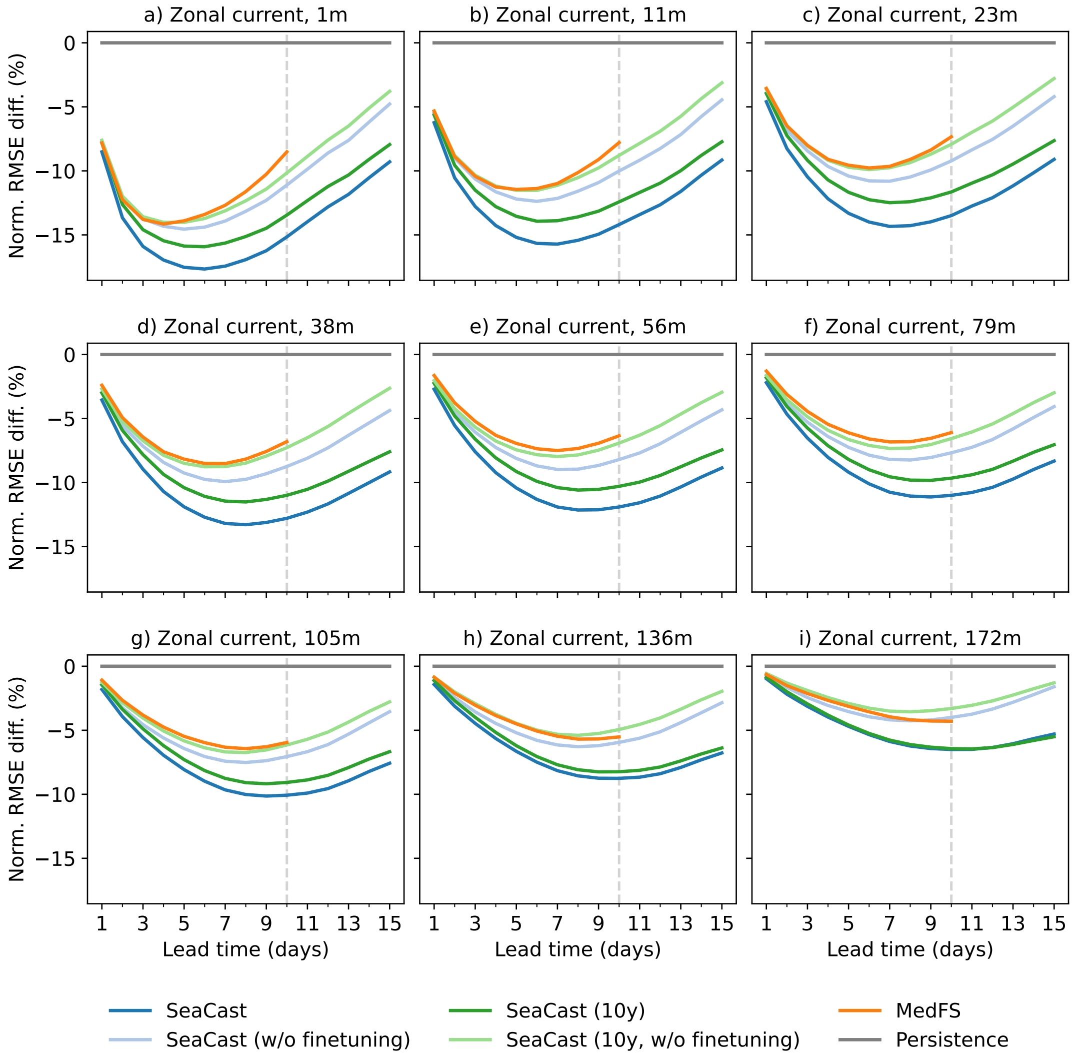


**Fig. S14** Normalized RMSE difference for zonal current as a function of lead time, comparing SeaCast models trained on different time spans against the persistence baseline.

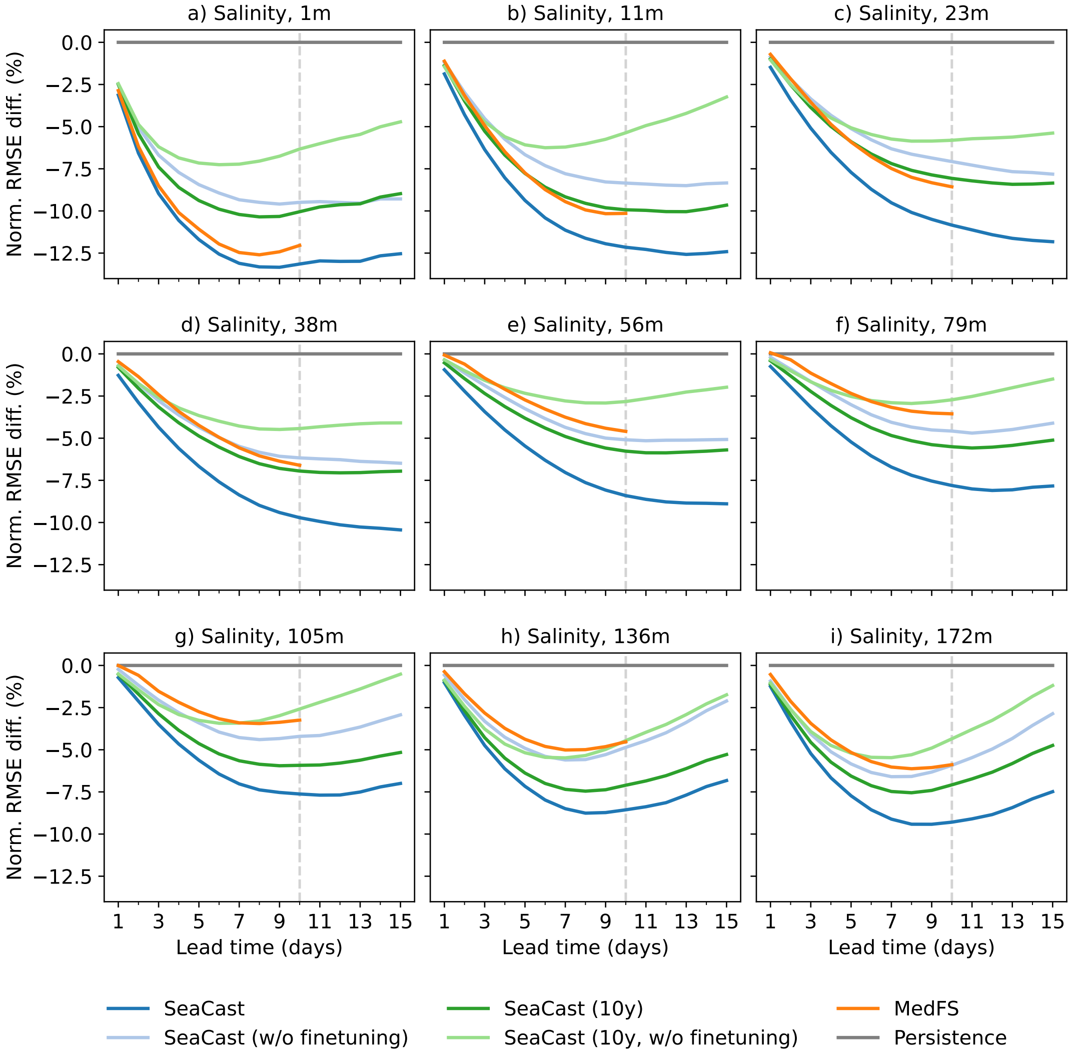


**Fig. S15** Normalized RMSE difference for salinity as a function of lead time, comparing SeaCast models trained on different time spans against the persistence baseline.

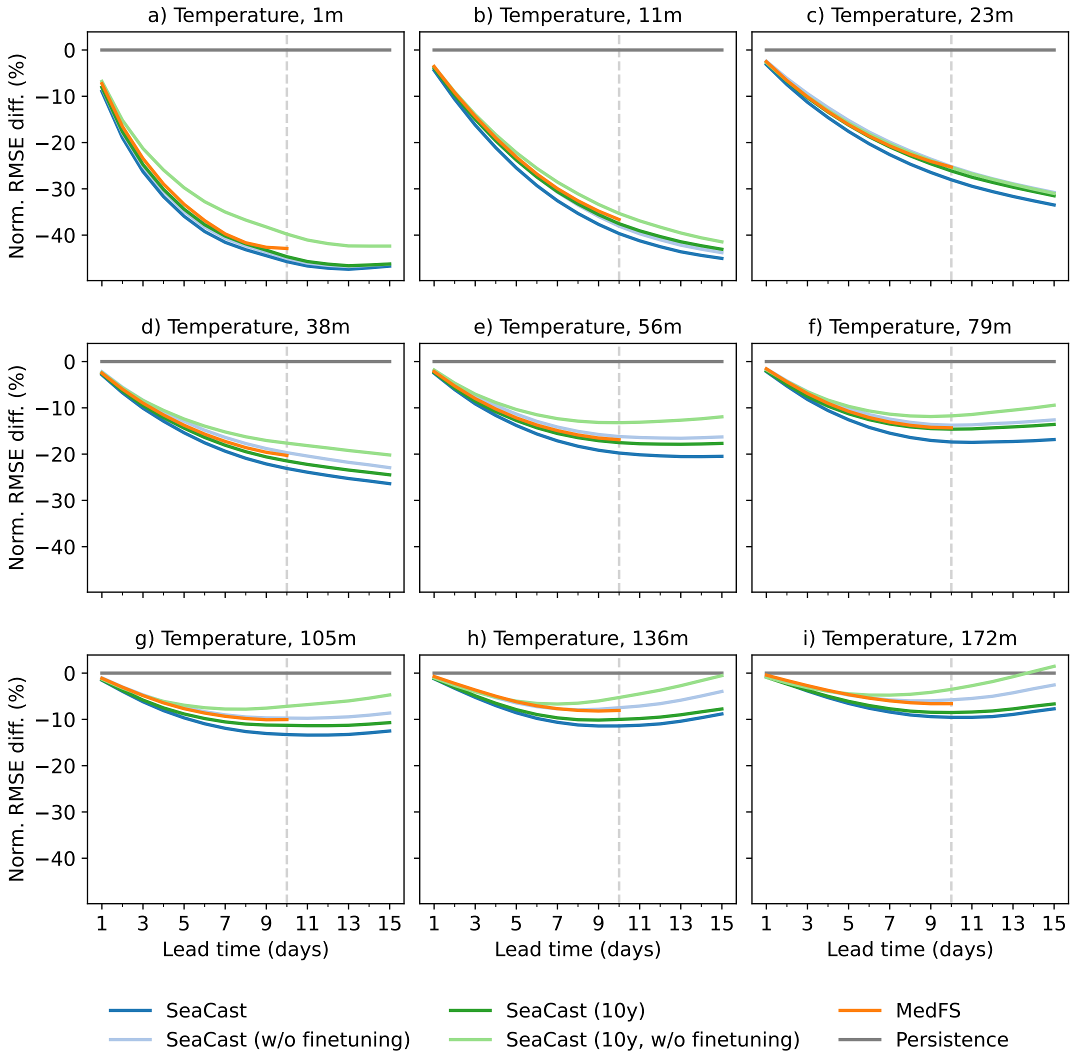


**Fig. S16** Normalized RMSE difference for temperature as a function of lead time, comparing SeaCast models trained on different time spans against the persistence baseline.

# E Example forecasts

To qualitatively illustrate SeaCast's forecasting capabilities, we show example outputs for a selection of fields. Forecasts are initialized from operational simulation states on October 1st, 2024, and include horizontal maps of currents, temperature, salinity at 11 m depth, as well as SSH. The bias is calculated towards the corresponding analysis fields.

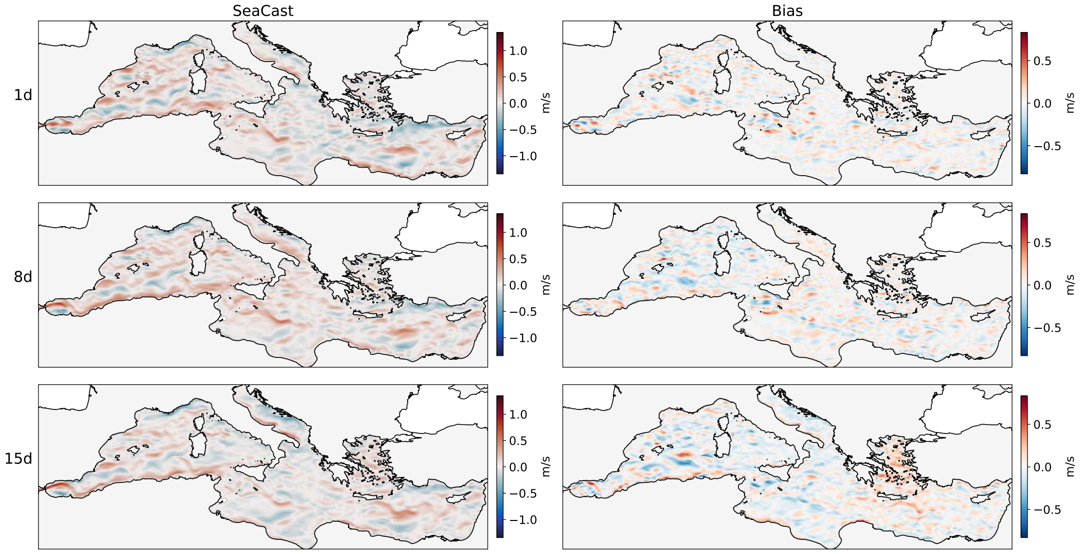


**Fig. S17** SeaCast forecast of zonal current at 11 m depth, initialized on October 1st, 2024.

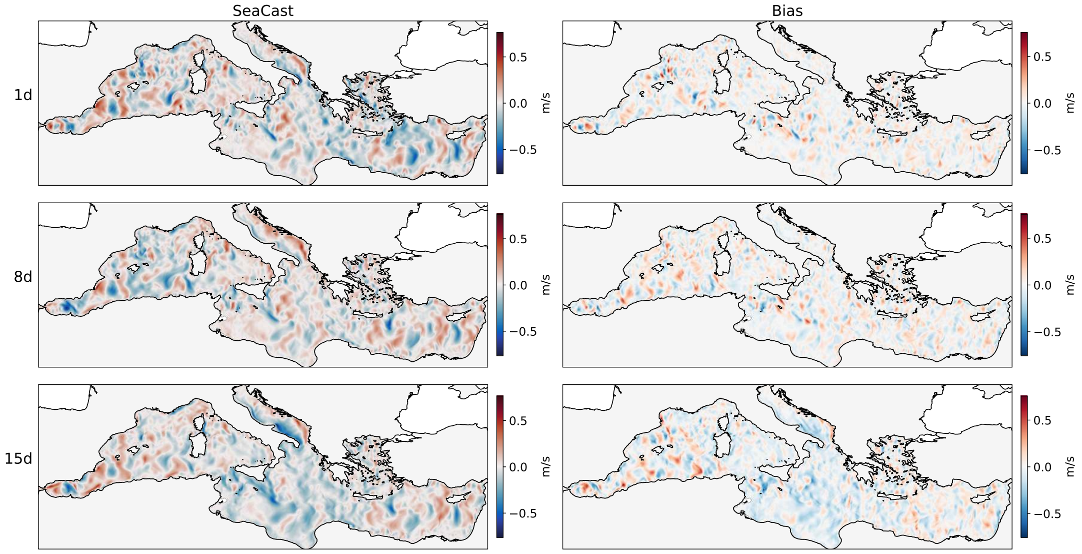


**Fig. S18** SeaCast forecast of meridional current at 11 m depth, initialized on October 1st, 2024.

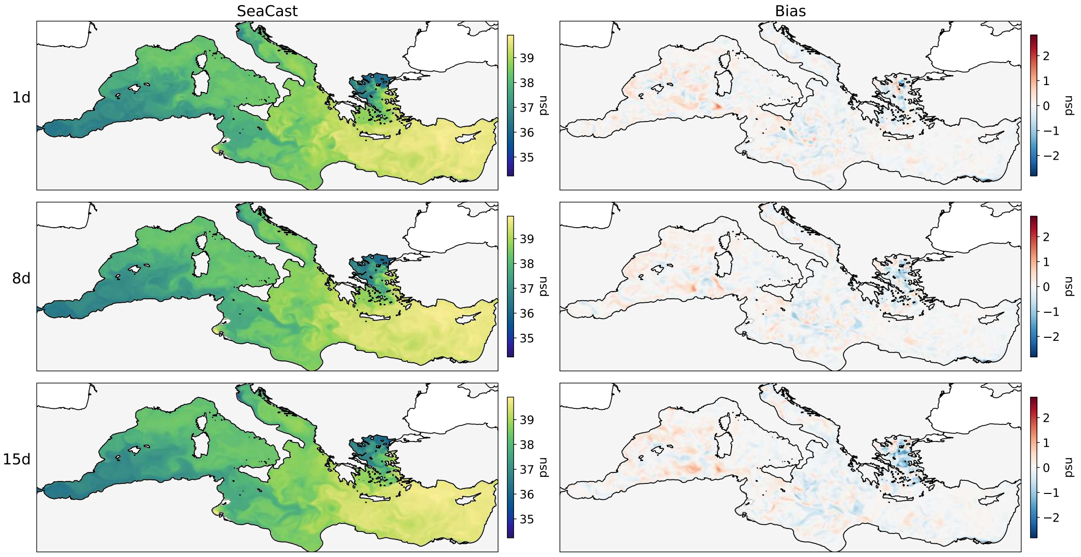


**Fig. S19** SeaCast forecast of salinity at 11 m depth, initialized on October 1st, 2024.

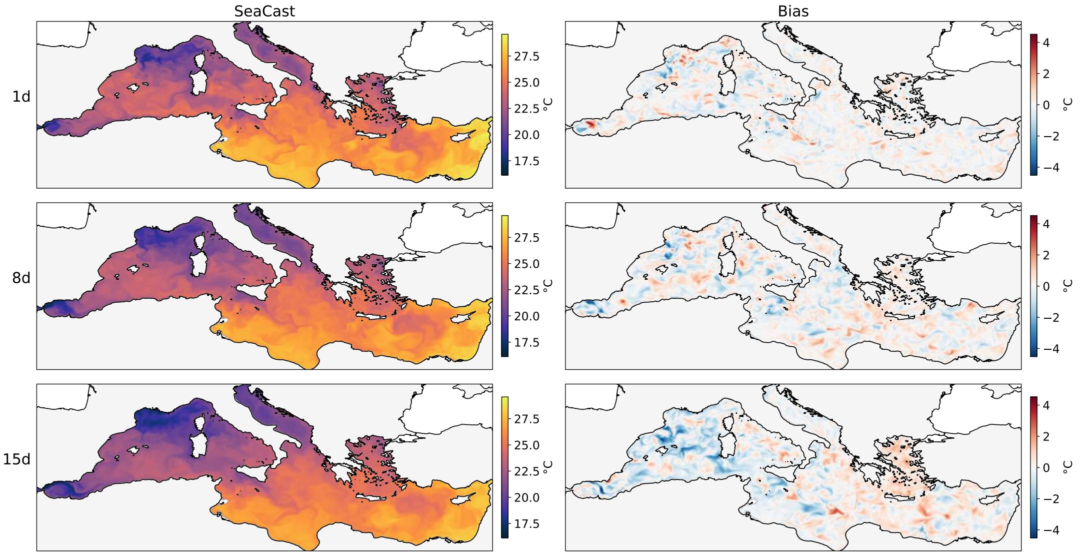


**Fig. S20** SeaCast forecast of temperature at 11 m depth, initialized on October 1st, 2024.

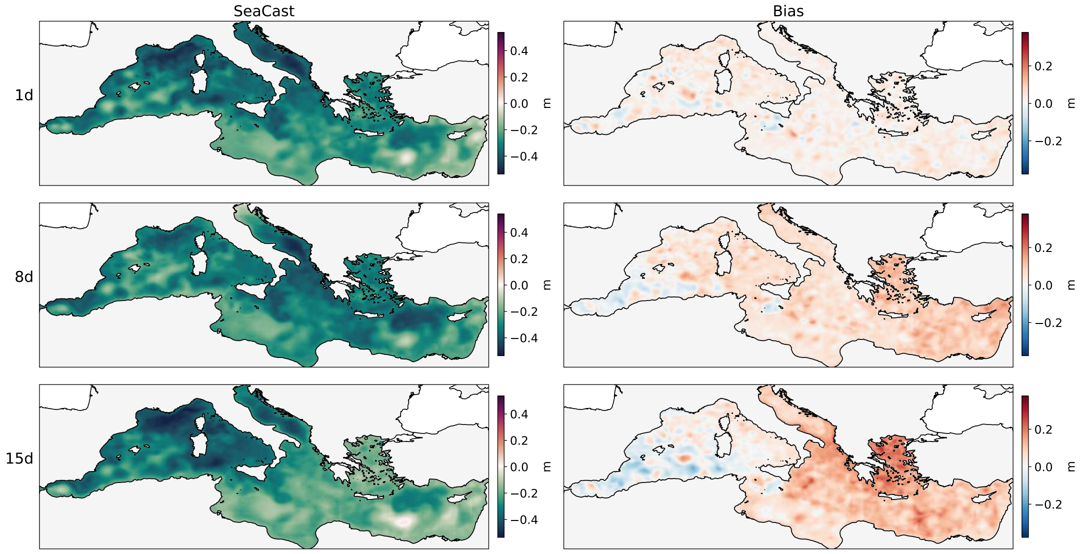


**Fig. S21** SeaCast forecast of SSH, initialized on October 1st, 2024.
